# Vitamin D Deficiency Aggravates Chronic Kidney Disease Progression after Ischemic Acute Kidney Injury

**DOI:** 10.1371/journal.pone.0107228

**Published:** 2014-09-15

**Authors:** Janaína Garcia Gonçalves, Ana Carolina de Bragança, Daniele Canale, Maria Heloisa Massola Shimizu, Talita Rojas Sanches, Rosa Maria Affonso Moysés, Lúcia Andrade, Antonio Carlos Seguro, Rildo Aparecido Volpini

**Affiliations:** Nephrology Department, University of São Paulo School of Medicine, São Paulo, Brazil; Nihon University School of Medicine, Japan

## Abstract

**Background:**

Despite a significant improvement in the management of chronic kidney disease (CKD), its incidence and prevalence has been increasing over the years. Progressive renal fibrosis is present in CKD and involves the participation of several cytokines, including Transforming growth factor-β1 (TGF-β1). Besides cardiovascular diseases and infections, several studies show that Vitamin D status has been considered as a non-traditional risk factor for the progression of CKD. Given the importance of vitamin D in the maintenance of essential physiological functions, we studied the events involved in the chronic kidney disease progression in rats submitted to ischemia/reperfusion injury under vitamin D deficiency (VDD).

**Methods:**

Rats were randomized into four groups: Control; VDD; ischemia/reperfusion injury (IRI); and VDD+IRI. At the 62 day after sham or IRI surgery, we measured inulin clearance, biochemical variables and hemodynamic parameters. In kidney tissue, we performed immunoblotting to quantify expression of Klotho, TGF-β, and vitamin D receptor (VDR); gene expression to evaluate renin, angiotensinogen, and angiotensin-converting enzyme; and immunohistochemical staining for ED1 (macrophages), type IV collagen, fibronectin, vimentin, and α-smooth mucle actin. Histomorphometric studies were performed to evaluate fractional interstitial area.

**Results:**

IRI animals presented renal hypertrophy, increased levels of mean blood pressure and plasma PTH. Furthermore, expansion of the interstitial area, increased infiltration of ED1 cells, increased expression of collagen IV, fibronectin, vimentin and α-actin, and reduced expression of Klotho protein were observed. VDD deficiency contributed to increased levels of plasma PTH as well as for important chronic tubulointerstitial changes (fibrosis, inflammatory infiltration, tubular dilation and atrophy), increased expression of TGF-β1 and decreased expression of VDR and Klotho protein observed in VDD+IRI animals.

**Conclusion:**

Through inflammatory pathways and involvement of TGF-β1 growth factor, VDD could be considered as an aggravating factor for tubulointerstitial damage and fibrosis progression following acute kidney injury induced by ischemia/reperfusion.

## Introduction

In most countries, the incidence and prevalence of chronic kidney disease (CKD) have been increasing over the years mainly due to the aging population and the presence of diabetic nephropathy [1], [2]. It is well established that acute kidney injury (AKI) after ischemia/reperfusion injury (IRI) is a major cause of AKI [3]. IRI physiology involves a complex interaction among vascular, tubular and inflammatory factors followed by a repair process that can restore function and epithelial differentiation or result in CKD with progressive development of fibrosis [4], [5].

It has been shown that the mortality of patients with CKD is directly related to renal function associated with cardiovascular diseases and infections [6]. However, such traditional risks explain only about half of mortality and various studies are being directed to non-traditional risk factors, such as vitamin D [6].

Vitamin D [25(OH)D] is a circulating hormone in the body indispensable for mineral homeostasis [7] and responsible for kidney protection and regulation of several physiological activities as well [8]. Thus, vitamin D deficiency (VDD) (<10 ng/mL) or insufficiency (10–30 ng/mL) can accelerate the progression of kidney disease [9]–[11]. The biologically active form of vitamin D is produced in the kidney by mitochondria of the renal proximal convoluted tubules, where 1α-hydroxylase converts 25-hydroxyvitamin D [25(OH)D] to 1,25-dihydroxyvitamin D3 [1,25 (OH)_2_D_3_] or calcitriol [12]. The classical 1,25 (OH)_2_D_3_ pathway requires the nuclear vitamin D receptor (VDR), which is a transcription factor for 1,25 (OH)_2_ D_3_ target genes [4], [5], [13].

The renal conversion of vitamin D into biologically active form is tightly regulated by several factors, including parathormone (PTH), phosphorus levels and fibroblast growth factor 23 (FGF-23) [12]. FGF-23 is a phosphatonin produced by osteocytes which promotes renal phosphate excretion [8], [14]–[16] and a close relationship between FGF-23 and Klotho is described [17]. The lack of Klotho gene expression (α-klotho) is associated with premature phenotypes related to aging as well as to hyperphosphatemia and low levels of vitamin D [18]. Klotho protein forms binary complexes with fibroblast growth factor receptors (FGFR), increasing Klotho affinity and selectivity for FGF-23 [17], playing an important role in vitamin D synthesis.

Given the importance of vitamin D in essential physiological functions and the respective low levels of this hormone observed in CKD, our aim was to study the vitamin D deficiency in a murine model of CKD progression after AKI induced by ischemia/reperfusion.

## Materials and Methods

### Experimental Animals and Induction of I/R Injury

The study was approved by the Research Ethics Committee of the University of São Paulo – School of Medicine, protocol numbered 088/12. All procedures were developed in strict accordance with local institutional guidelines and with well-established international standards for the care and use of laboratory animals. All surgery was performed under appropriate anesthesia, and all efforts were made to minimize suffering.

Male Wistar rats (180–200 g) were provided by the University of São Paulo - School of Medicine animal facility. During the 90-day experiment, rats were maintained under standard laboratory conditions, receiving vitamin D-free or standard diets (MP Biomedicals, Irvine, CA, USA) and free access to tap water for 90 days. Rats were divided into four groups: (C) Control (n = 11), received a standard diet for 90 days and submitted to sham surgery; (VDD) Vitamin D Deficiency (n = 8), received a vitamin D-free diet for 90 days and submitted to sham surgery; (IRI) Ischemia/Reperfusion Injury (n = 10), received a standard diet for 90 days and submitted to ischemia/reperfusion injury; and (VDD+IRI) Vitamin D Deficiency plus Ischemia/Reperfusion Injury (n = 9), received a vitamin D-free diet for 90 days and submitted to ischemia/reperfusion injury.

#### Sham Surgery

On day 28, rats from Control and VDD groups were anesthetized with 2,2,2-Tribromoethanol (250 mg/Kg body weight), and a suprapubic incision was made and then sutured immediately.

#### Induction of Ischemia/Reperfusion Injury

As described above, on day 28, rats from IRI and VDD+IRI groups were anesthetized, a suprapubic incision was made for induction of ischemic renal injury by clamping both renal arteries for 45 min, followed by reperfusion.

### Analysis of Urine Samples

On day 89, all rats were placed in individual metabolic cages, on a 12/12-h light/dark cycle, with free access to drinking water. We collected 24-h urine samples, centrifuged them and analyzed the supernatants. We measured urinary concentrations of sodium and calcium by ion-selective electrodes (ABL800 Flex Analyzer – Radiometer, Brønshøj, Denmark) and urinary phosphorus by automated enzymatic colorimetric assay (COBAS C111 Analyzer – Roche, USA). Urinary protein excretion was measured by a colorimetric system using a commercial kit (Labtest Diagnóstica, Minas Gerais, Brazil).

### Inulin Clearance and Hemodynamic Studies

On day 90, the animals were anesthetized with sodium thiopental (50 mg/Kg BW) and placed on a temperature-regulated surgical table. The trachea was cannulated (PE-240 catheter) and spontaneous breathing was maintained. The jugular vein was cannulated (PE-60 catheter) for infusion of inulin and fluids. To monitor mean arterial pressure (MAP) and collect blood samples, the right carotid artery was catheterized with a PE-50 catheter. We assessed MAP with a data acquisition system (MP100; Biopac Systems, Santa Barbara, CA, USA). To collect urine samples, the urinary bladder was cannulated (PE-240 catheter) by suprapubic incision. After completion of the cannulation surgical procedure, a loading dose of inulin (100 mg/Kg body weight diluted in 1 mL of 0.9% saline) was administered through the jugular vein. Subsequently, a constant infusion of inulin (10 mg/kg body weight in 0.9% saline) was started and continued at 0.04 mL/min throughout the whole experiment. Three urine samples were collected at 30-min intervals. Blood samples were obtained at the beginning and at the end of the experiment. Inulin clearance values represent the mean of three periods. Blood and urine inulin were determined by the anthrone method, and the glomerular filtration rate (GFR) data is expressed as ml/min/100 g BW. To measure the renal blood flow (RBF), an ultrasonic flow probe was placed around the exposed renal artery (T402; Transonic Systems, Bethesda, MD, USA) and RBF was expressed as ml/min. To calculate renal vascular resistance (RVR, in mmHg/ml/min), we divided blood pressure by RBF.

### Evaluation of Biochemical Parameters

To assess plasma levels of 25-hydroxyvitamin D [25(OH)D], parathormone (PTH), fibroblast growth factor 23 (FGF-23), sodium (P_Na_), potassium (P_K_), phosphate (P_P_) and calcium (P_Ca_), we collected blood samples after the clearance studies. We assessed 25(OH)D by radioimmunoassay (RIA) using a commercial kit (25-Hydroxyvitamin D^125^, DiaSorin, Vercelli, Italy) and PTH by Enzyme-Linked Immunosorbet Assay (ELISA) using a commercial kit (Rat Bioactive Intact PTH, Immunotopics, CA, USA). FGF-23 levels were assessed by ELISA using a commercial kit (FGF-23 ELISA Kit, Kainos Laboratories, Tokyo, Japan). P_P_ and P_Ca_ were evaluated by automated enzymatic colorimetric assay (COBAS C111 Analyzer – Roche, USA) while P_Na_ and P_K_ were measured by ion-selective electrodes (ABL800 Flex Analyzer – Radiometer, Brønshøj, Denmark).

### Tissue Sample Collection/Preparation

After blood samples collection, we perfused kidneys with phosphate-buffered solution (PBS, pH 7.4). Right kidneys were frozen in liquid nitrogen and stored at −80°C for Western blotting and real-time quantitative polymerase chain reaction (qPCR). Left kidneys were removed and weighed. Fragments of left kidney were fixed in 10% neutral-buffered formalin or methacarn's solution for 24 hours and in 70% alcohol thereafter. Kidney blocks were embedded in paraffin and cut into 4-µm sections for histological and immunohistochemical examination.

### Preparation of Samples for Western Blotting

Kidney samples were homogenized in ice-cold isolation solution (200 mM manitol, 80 mM HEPES and 41 mM KOH, pH 7.5) containing a protease inhibitors cocktail (Sigma Chemical Company, St. Louis, MO, USA) using a homogenizer (Polytron - PT 10–35, Brinkmann Instruments, Westbury, NY, USA). Homogenates were centrifuged at low speed (2000×g) for 15 min at 4°C to remove nuclei and cell debris. Supernatants were isolated, and protein was determined by Bradford assay (BioAgency Laboratórios, São Paulo, Brazil).

### Electrophoresis and Immunoblotting

Proteins were separated on SDS-polyacrylamide minigels by electrophoresis [19]. After transfer by electroelution to polyvinylidene difluoride (PVDF) membranes (GE Healthcare Limited, Little Chalfont, UK), blots were blocked for 60 minutes with 5% non-fat dry milk in Tris-buffered saline solution. Blots were then incubated overnight with antibodies against Actin (1∶5,000), VDR (1∶500), Klotho (1∶500) and TGF-beta (1∶200) (Santa Cruz Biotechnology, CA, USA). The labeling was visualized with horseradish peroxidase-conjugated secondary antibody (anti-rabbit IgG, diluted 1∶2,000, or anti-goat, diluted 1∶10,000, Sigma Chemical, St. Louis, MO, USA) and enhanced chemiluminescence (ECL) detection system (GE Healthcare Limited, Little Chalfont, UK).

#### Kidney protein levels

The images were obtained using an imaging system (Alliance 4.2; Uvitec, Cambridge, UK). We used densitometry to quantitatively analyze the protein levels, normalizing the bands to actin expression.

### Gene Expression

We performed real-time qPCR in frozen renal tissue, assessing the following genes: renin (Rn00561847_m1), angiotensinogen (Rn00593114_m1), and angiotensin converting enzyme (Rn00561094_m1), (Applied Biosystems, Foster City, CA, USA). We extracted and prepared total RNA. For cDNA synthesis, we used total RNA and a Superscript VILO MasterMix (Invitrogen Technologies, Carlsbad, CA, USA). We performed real-time PCR using TaqMan on Step One Plus (Applied Biosystems, Foster City, CA, USA). All primers were purchased from Invitrogen. Relative gene expression values were evaluated with the 2^-ΔΔCt^ method [20] using GAPDH (Rn01775763_g1) as housekeeping gene.

### Light Microscopy

Four-µm histological sections of kidney tissue were stained with Masson's trichome and examined under light microscope. The fractional interstitial area (FIA) of the renal cortex was determined by morphometry with a light camera connected to an image analyzer (Axiovision, Carl Zeiss, Eching, Germany). We analyzed 30 grid fields (0.087 mm^2^ each) per kidney cortex. The interstitial areas were demarcated manually on a video screen, and the proportion of the field they occupied was determined by computerized morphometry [21].The morphometric examination was blinded to minimize observer bias, i.e. the observer was unaware of the treatment group from which the tissue originated.

### Immunohistochemistry

Samples were processed in 4-µm paraffin sections and then subjected to incubation overnight at 4°C according to each protocol developed for each primary antibody. We used the following antibodies: (1∶500) monoclonal anti-ED1 (macrophages) (AbD Serotec, Oxford, UK); (1∶200) polyclonal anti-Collagen IV (Abcam, Cambridge, UK); (1∶400) polyclonal anti-FN1 (fibronectin) (Sigma-Aldrich, Saint Louis, MO, USA); (1∶1,000) monoclonal anti-α-smooth-muscle actin (α-SMA) (Millipore, Billerica, MA, USA); and (1∶50) monoclonal anti-vimentin (Dako, Glostrup, Denmark). The reaction product was detected with an avidin-biotin-peroxidase complex (Vector Laboratories, Burlingame, CA, USA). The color reaction was developed with 3,3-diaminobenzidine (Sigma Chemical, St. Louis, MO, USA) in the presence of hydrogen peroxide, and the sections were counterstained with Harris' hematoxylin.

For ED1, we analyzed 40–60 renal cortex fields. The result of the immunoreaction was quantified by couting the number of ED1 positive cells per field (0.087 mm^2^ each) and averaging the number of cells per field for each section [21]. To evaluate immunoreactivity to collagen IV, fibronectin, α-SMA and vimentin, the volume ratios of positive areas of renal tissue sections, determined by the color limit, were obtained using Image-Pro Plus software (Media Cybernetics, Silver Spring, MD, USA) and the results were expressed as percentages [22].

### Statistical Analysis

All quantitative data are mean±standard error of the mean (SEM). Comparisons between groups were made by unpaired t-test. Comparisons among groups were made by one-way analysis of variance followed by the Student–Newman–Keuls test. Values of p<0.05 were considered statistically significant.

## Results

### Body weight and hemodynamics

As described in Table 1, there were no differences in body weight among the studied groups since all animals showed similar food ingestion (∼25 g/day) during 90 days. The kidney weight/body weight ratio was calculated and, as expected, IRI and VDD+IRI groups presented an increase of this proportion (Table 1). We did not observe differences among the groups concerning glomerular filtration rate (GFR), renal blood flow (RBF) and renal vascular resistance (RVR). However, we found increased levels (mmHg) of mean arterial pressure (MAP) in VDD (135.2±3.4), IRI (137.3±3.6) and VDD+IRI (134.5±5.2) when compared to Control (119.4±2.3) group (p<0.05), Table 1.

**Table 1:** Body weight, kidney weight, renal function and hemodynamic measurements after 90 days evaluated in Control rats (C), Vitamin D deficient rats (VDD), rats submitted to Ischemia/Reperfusion Injury (IRI), and Vitamin D deficient rats submitted to Ischemia/Reperfusion Injury (VDD+IRI).

	C	VDD	IRI	VDD+IRI
BW	519.4±11.6	551.4±19.8	515.8±13.6	510.1±14.1
KW/BW	0.36±0.01	0.35±0.01	0.42±0.02 ^c,f^	0.44±0.01 ^c,e^
GFR	0.62±0.02	0.56±0.04	0.57±0.04	0.57±0.02
MAP	119.4±2.3	135.2±3.4 ^c^	137.3±3.6 ^c^	134.5±5.2 ^c^
RBF	5.61±0.02	5.82±0.06	5.62±0.08	5.83±0.07
RVR	22.02±0.52	24.82±1.09	24.72±0.67	23.4±0.91

BW, body weight (g); KW/BW. kidney weight/body weight ratio; GFR, inulin clearance (mL/min/100 g); MAP, mean arterial pressure (mmHg); RBF, renal blood flow (mL/min); RVR, renal vascular resistance (mmHg/mL/min). Values are mean ± SEM. ^c^ p<0.05 vs. C; ^e^ p<0.01 vs. VDD; ^f^ p<0.05 vs. VDD.

### Vitamin D and PTH levels

The animals were maintained on a standard or a free-vitamin D diet for 90 days. Over the experimental period, the levels of 25(OH)D from VDD and VDD+IRI groups were undetectable (<1.5 ng/mL), while Control and IRI groups presented 15.4 and 15.0 ng/mL respectively (Table 2). Concerning PTH data, we found significant higher levels (pg/mL) of the respective hormone in VDD (1,250±155) and VDD+IRI (2,187±336), showing the negative feedback caused by vitamin D deficiency in these groups. Both VDD and VDD+IRI groups presented a significant increase of PTH levels when compared to Control (318±59) and IRI (453±96) groups. Furthermore, VDD+IRI presented significant high levels of PTH when compared to the other groups (Table 2).

**Table 2:** Biochemical parameters after 90 days evaluated in Control rats (C), Vitamin D deficient rats (VDD), rats submitted to Ischemia/Reperfusion Injury (IRI), and Vitamin D deficient rats submitted to Ischemia/Reperfusion Injury (VDD+IRI).

	C	VDD	IRI	VDD+IRI
25(OH)D	15.4±1.0	<1.5 (undetectable) ^a^	15.0±0.6 ^d^	<1.5 (undetectable)^a,g^
PTH	318±59	1,250±155 ^c^	453±96 ^f^	2,187±336 ^a,f,g^
Aldosterone	165.7±17.2	455.8±69.0 ^c^	416.0±89.2 ^c^	502.5±134.2 ^c^
FGF-23	293.1±33.8	41.8±14.6 ^a,g^	225.4±24.0	77.4±18.5 ^a,g^
P_P_	4.8±0.2	5.2±0.2	5.5±0.2	5.3±0.3
P_Ca_	9.48±0.50	8.81±0.41	9.28±0.15	8.17±0.40
P_Na_	139.5±1.2	136±0.6	137.4±2.2	137.7±0.6
P_K_	4.6±0.1	4.0±0.2	4.5±0.1	4.3±0.2
UV	14.23±0.93	13.40±1.03	14.42±1.71	11.13±1.77
U_Prot_V	5.97±0.53	17.16±1.56 ^a^	23.97±1.43 ^a,d^	32.31±0.52 ^a,d,g^

25(OH)D, 25 hydroxyvitamin D (ng/mL); PTH, parathormone (pg/mL); Aldosterone (pg/mL); FGF-23, fibroblast growth factor 23 (pg/mL); P_P_, plasma phosphate concentration (mg/dL); P_Ca_, plasma calcium concentration (mg/dL); P_Na_, plasma sodium concentration (mEq/L); P_K_, plasma potassium concentration (mEq/L); UV, urinary volume (mL/24 h); U_Prot_V, urinary protein excretion (mg/24 h). Values are means ± SEM. ^a^ p<0.001, ^b^ p<0.01, ^c^ p<0.05 vs. C; ^d^ p<0.001 vs. VDD; ^f^ p<0.05 vs. VDD; ^g^ p< 0.001 vs. IRI.

### Vitamin D deficiency and blood pressure control

In order to further investigate the role of vitamin D on blood pressure control, we evaluated the gene expression (qPCR) of some renin-angiotensin system (RAS) compounds and plasma aldosterone levels as well. The qPCR results showed increased gene expression of renin, angiotensinogen (AGT) and angiotensin converting enzyme (ACE) in VDD, IRI and VDD+IRI when compared to control group (Table 3). In addition, we found higher levels of aldosterone (pg/mL) in VDD (455.8±69.0), IRI (416.0±89.2) and VDD+IRI (502.5±134.2) when compared to Control (165.7±17.2) group (Table 2). Taken together, our results reinforce the role of vitamin D on blood pressure control.

**Table 3:** Relative gene expression of α-klotho and renin-angiotensin system (RAS) compounds after 90 days evaluated in Control rats (C), Vitamin D deficient rats (VDD), rats submitted to Ischemia/Reperfusion Injury (IRI), and Vitamin D deficient rats submitted to Ischemia/Reperfusion Injury (VDD+IRI).

	C	VDD	IRI	VDD+IRI
renin	0.68±0.49	2.28±0.28 ^b^	1.85±0.47 ^c^	2.34±0.28 ^c^
AGT	0.95±0.45	2.95±0.69 ^c^	4.02±0.60 ^b^	4.53±0.63 ^b^
ACE	1.10±0.63	3.94±0.82 ^c^	5.28±0.94 ^c^	5.90±1.28 ^c^
α-klotho	3.27±1.15	2.07±0.41	2.39±0.38	1.74±0.46

AGT, angiotensinogen; ACE, angiotensin-converting enzyme. Values are mean ± SEM. Relative gene expression values were evaluated with the 2^-ΔΔCt^ method using GAPDH as housekeeping gene. ^b^ p<0.01 and ^c^ p<0.05 vs. C.

### Proteinuria, calcium, phosphorus and FGF-23 – Klotho axis

**Figure 1:**
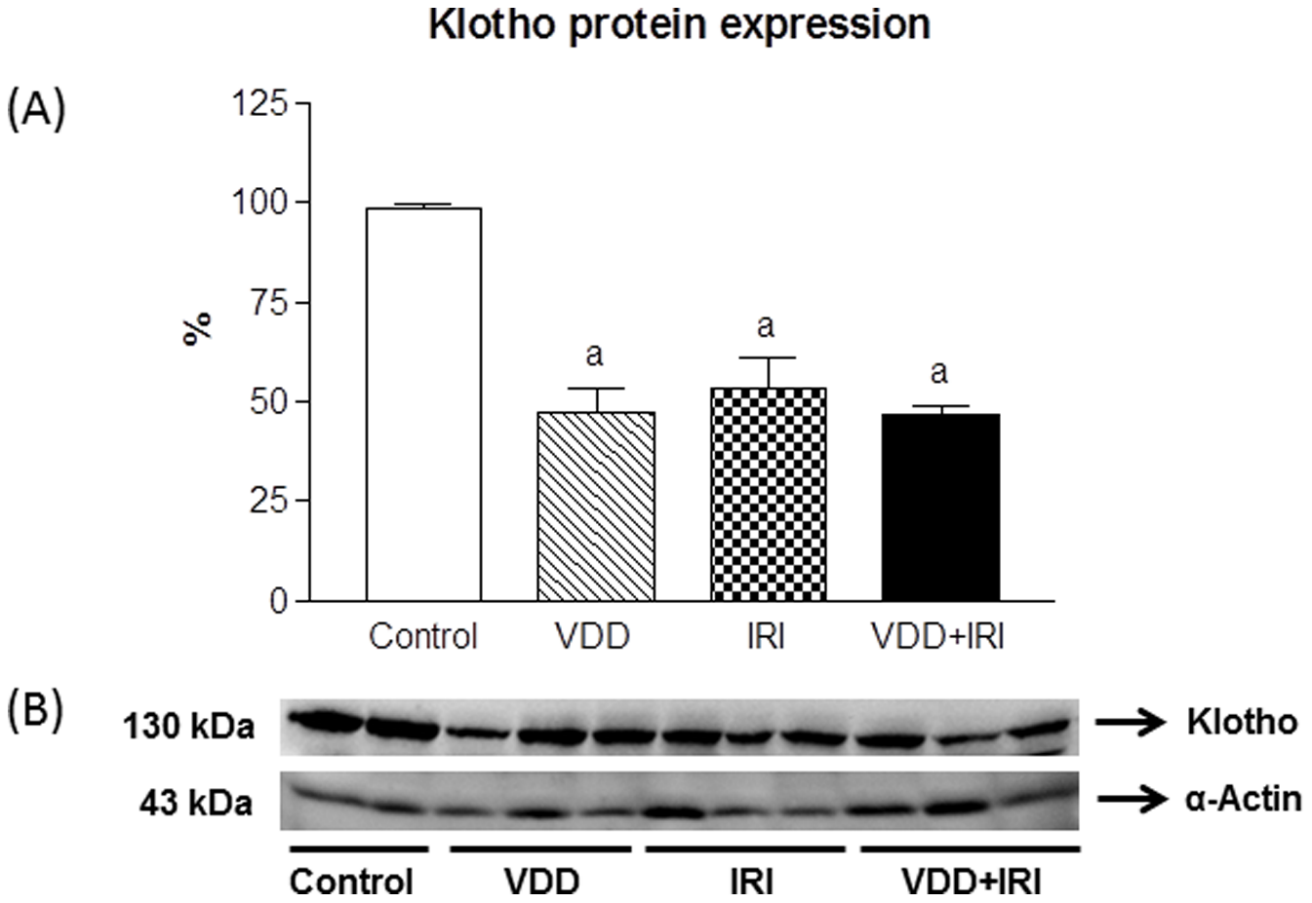
Semiquantitative immunoblotting of kidney fractions. (A) A densitometric analysis of samples from control (n = 4), VDD (n = 6), IRI (n = 6) and VDD+IRI (n = 6) rats is shown. (B) Immunoblots reacted with anti-Klotho revealing a 130-kDa band. Values are mean ± SEM. ^a^ p<0.001 vs. C.

As shown in Table 2, we observed a progressive and significant (p<0.001) increase of proteinuria (mg/24 h) among the studied groups: Control (5.96±0.53), VDD (17.16±1.56), IRI (23.97±1.43) and VDD+IRI (32.31±0.51). Neither calcium and phosphorus levels nor urinary volume presented differences among the groups (Table 2). Considering the fact that the levels of calcium and phosphorus are closely related to FGF-23 - Klotho axis, we investigated plasmatic levels of FGF-23 and the renal expression of Klotho. We observed decreased levels of FGF-23 (pg/mL) in VDD (41.8±14.6) and VDD+IRI (77.4±18.5) groups when compared to Control (293.1±33.8) and IRI (225.4±24.0) groups (Table 2). Also, we found a decreased expression of Klotho protein (%) in VDD (47.5±6.0), IRI (53.3±7.6) and VDD+IRI (46.7±2.5) when compared to Control (98.8±0.8) group (Figure 1). In addition, similar profile was found concerning gene expression of α-klotho (Table 3).

### Histomorphological studies

**Figure 2:**
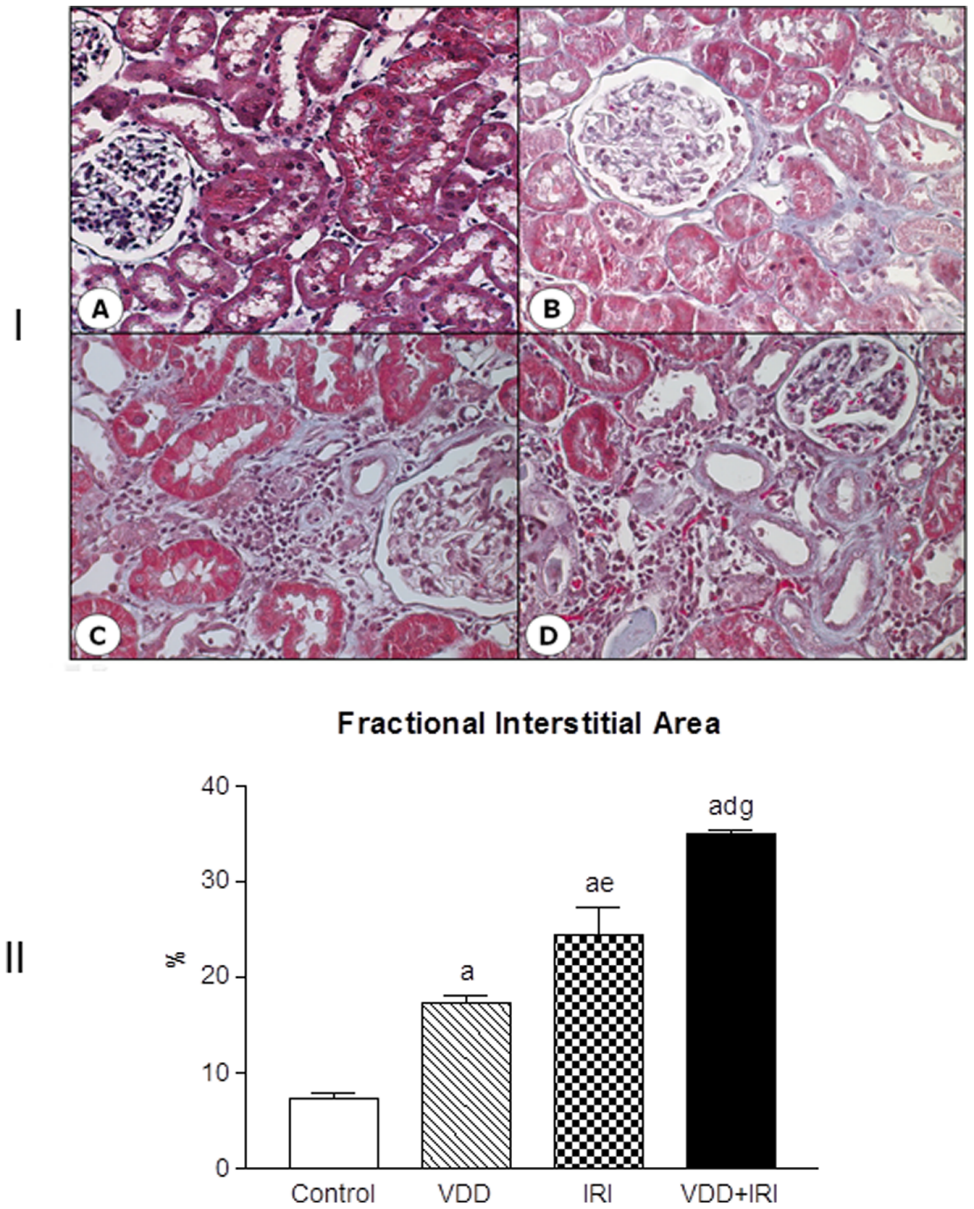
[I] Representative photomicrographs of renal histological changes after 90 days observed in a control rat (A), a vitamin D deficient rat (B), in a rat submitted to ischemia/reperfusion injury (C), and in a vitamin D deficient rat submitted to ischemia/reperfusion injury (D). [II] Fractional interstitial area evaluated 90 days after in Control (C), Vitamin D Deficiency (VDD), Ischemia-Reperfusion Injury (IRI), and Vitamin D Deficiency and Ischemia/Reperfusion Injury (VDD+IRI) groups. Values are mean ± SEM. ^a^ p<0.001 vs. C; ^d^ p<0.001 vs. VDD; ^e^ p<0.01 vs. VDD; ^g^ p<0.001 vs. IRI.

Light microscopy studies revealed histological alterations such as interstitial fibrosis, tubular atrophy and dilatation, and inflammatory cell infiltrates in the renal cortex of vitamin D deficient and ischemic/reperfusion injury groups (Figure 2). The alterations were subtle, but evident in VDD animals and more prominent in IRI and VDD+IRI groups. In addition, morphometric studies were performed to evaluate the fractional interstitial area (FIA) of the renal cortex. We observed a progressive and increasing involvement of the tubulointerstitial compartment, featuring interstitial expansion and renal fibrosis as follows: Control and VDD groups showed 7.35% and 17.23% of FIA, respectively while IRI and VDD+IRI groups presented 24.41 and 34.87% of FIA, respectively. Based on our results, we could infer that vitamin D deficiency exerts an important role on interstitial expansion and fibrosis formation.

### Macrophages infiltration

**Figure 3:**
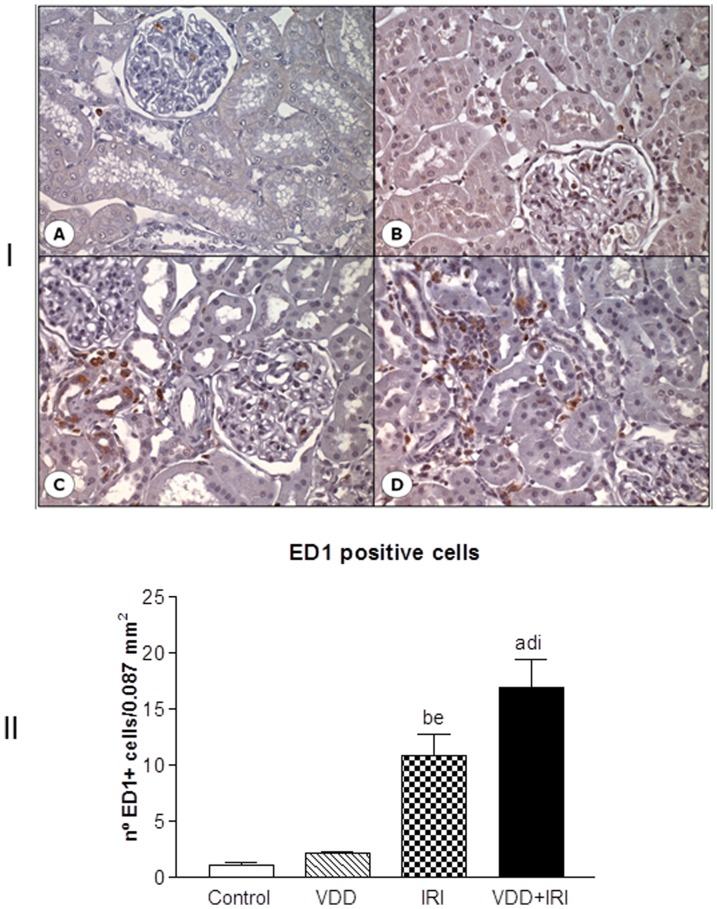
[I] Expression of ED1 positive cells in the renal cortex of a control rat (A), a vitamin D deficient rat (B), in a rat submitted to ischemia/reperfusion injury (C), and in a vitamin D deficient rat submitted to ischemia/reperfusion injury (D). [II] Number of ED1 positive cells per field (0.087 mm^2^) evaluated 90 days after in Control (C), Vitamin D Deficiency (VDD), Ischemia-Reperfusion Injury (IRI), and Vitamin D Deficiency and Ischemia/Reperfusion Injury (VDD+IRI) groups. Values are mean ± SEM. ^a^ p<0.001 vs. C; ^b^ p<0.01 vs. C; ^d^ p<0.001 vs. VDD; ^e^ p<0.01 vs. VDD; ^i^ p<0.05 vs. IRI.

The number of ED1 positive cells, a marker to macrophages, was evaluated by immunohistochemical studies. We found a significant number of infiltrating ED1 positive cells/per field (0.087 mm^2^) in IRI (10.92±1.89) and VDD+IRI (16.93±2.49) when compared to VDD (2.18±0.14) and Control (1.14±0.19) groups. Vitamin D deficiency enhanced this alteration since VDD+IRI presented an increased number of ED1 positive cells than did the other groups (Figure 3).

### Extracellular matrix components

**Figure 4:**
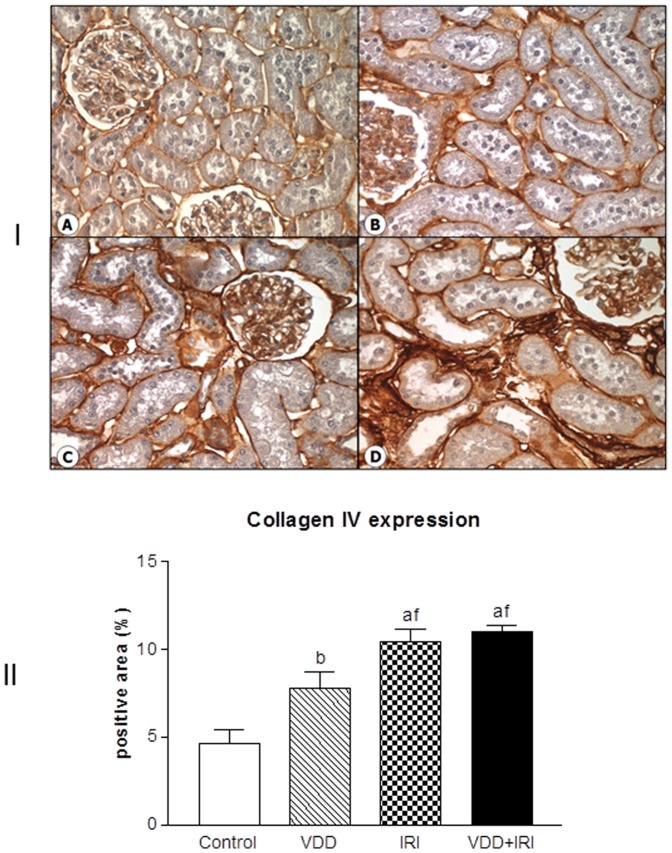
[I] Expression of type IV collagen in the renal cortex of a control rat (A), a vitamin D deficient rat (B), in a rat submitted to ischemia/reperfusion injury (C), and in a vitamin D deficient rat submitted to ischemia/reperfusion injury (D). [II] Evaluation of type IV collagen expression 90 days after in Control (C), Vitamin D Deficiency (VDD), Ischemia-Reperfusion Injury (IRI), and Vitamin D Deficiency and Ischemia/Reperfusion Injury (VDD+IRI) groups. Values are mean ± SEM. ^a^ p<0.001 vs. C; ^b^ p<0.01 vs. C; ^f^ p<0.05 vs. VDD.

**Figure 5:**
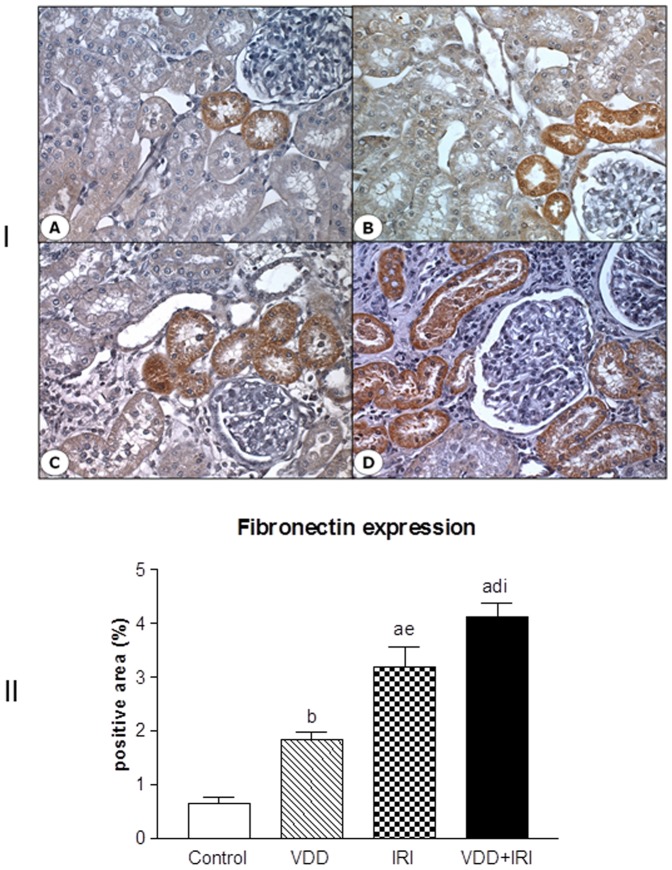
[I] Expression of fibronectin in the renal cortex of a control rat (A), a vitamin D deficient rat (B), in a rat submitted to ischemia/reperfusion injury (C), and in a vitamin D deficient rat submitted to ischemia/reperfusion injury (D). [II] Evaluation of fibronectin expression 90 days after in Control (C), Vitamin D Deficiency (VDD), Ischemia-Reperfusion Injury (IRI), and Vitamin D Deficiency and Ischemia/Reperfusion Injury (VDD+IRI) groups. Values are means ± SEM. ^a^ p<0.001 vs. C; ^b^ p<0.01 vs. C; ^d^ p<0.001 vs. VDD; ^e^ p<0.01 vs. VDD; ^i^ p<0.05 vs. IRI.

As described above, our histological results showed that vitamin D deficiency is an aggravating factor for the expansion of tubulointerstitial compartment and fibrosis formation. This process is complex and involves the production and secretion of many extracellular matrix (ECM) components. So, we performed immunohistochemical studies for type IV collagen and fibronectin, two fibrous components of ECM. Our results showed increased expression of type IV collagen (Figure 4) and fibronectin (Figure 5), demonstrated as percentage of positive area, in the renal cortex of VDD, IRI and VDD+IRI groups when compared to Control group. Vitamin D deficiency enhanced the immunostainings for both ECM markers (Figures 4 and 5).

### Phenotypic alteration of renal cells

**Figure 6:**
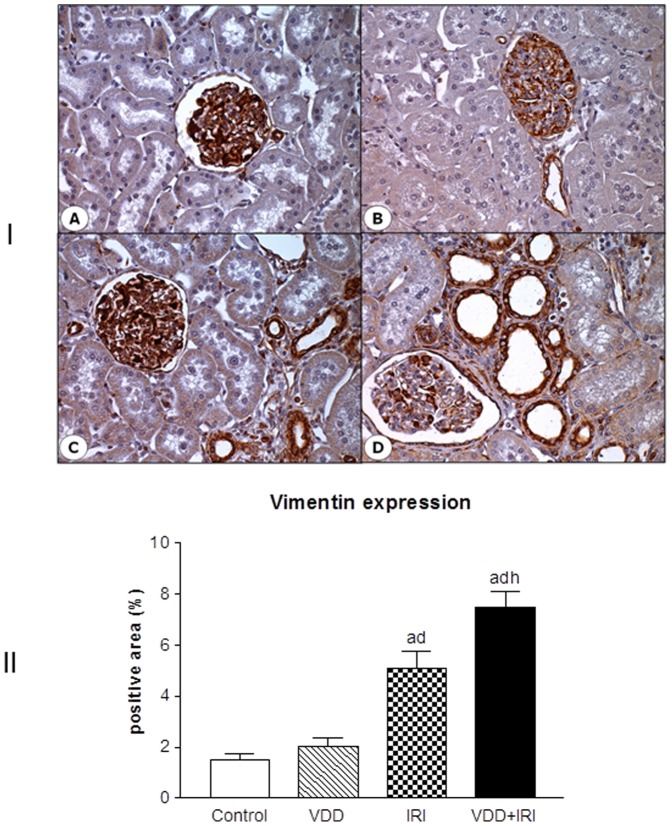
[I] Expression of vimentin in the renal cortex of a control rat (A), a vitamin D deficient rat (B), in a rat submitted to ischemia/reperfusion injury (C), and in a vitamin D deficient rat submitted to ischemia/reperfusion injury (D). [II] Evaluation of vimentin expression 90 days after in Control (C), Vitamin D Deficiency (VDD), Ischemia-Reperfusion Injury (IRI), and Vitamin D Deficiency and Ischemia/Reperfusion Injury (VDD+IRI) groups. Values are mean ± SEM. ^a^ p<0.001 vs. C; ^d^ p<0.001 vs. VDD; ^h^ p<0.01 vs. IRI.

**Figure 7:**
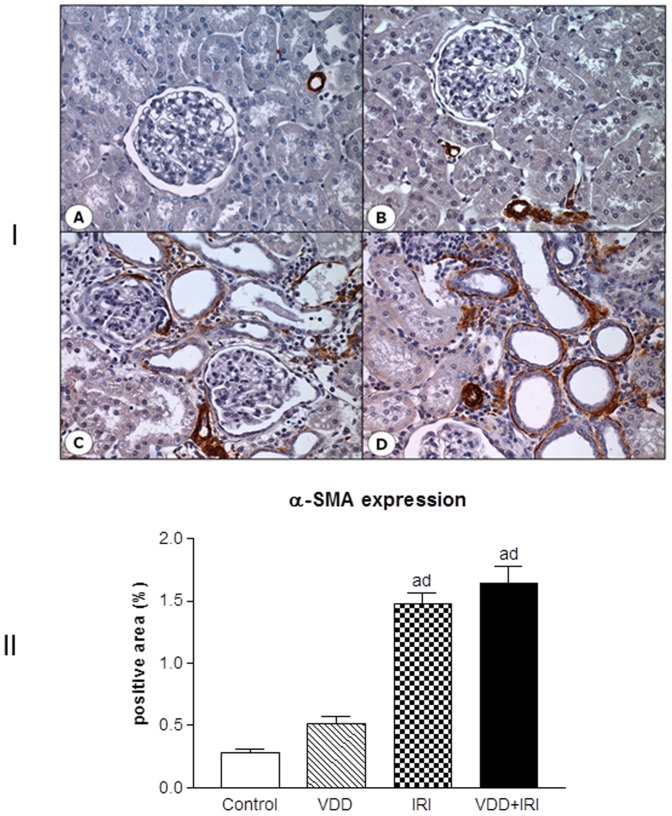
[I] Expression of α-SMA in the renal cortex of a control rat (A), a vitamin D deficient rat (B), in a rat submitted to ischemia/reperfusion injury (C), and in a vitamin D deficient rat submitted to ischemia/reperfusion injury (D). [II] Evaluation of α-SMA expression 90 days after in Control (C), Vitamin D Deficiency (VDD), Ischemia-Reperfusion Injury (IRI), and Vitamin D Deficiency and Ischemia/Reperfusion Injury (VDD+IRI) groups. Values are mean ± SEM. ^a^ p<0.001 vs. C; ^d^ p<0.001 vs. VDD.

Besides ECM markers studies, we also aimed to evaluate the presence of phenotypic alteration of renal tubular cells. We used the expression of vimentin to detect tubular injury and α-smooth muscle actin (α-SMA) as a marker for interstitial fibroblast activation. In Control group, the expression of vimentin and α-SMA was identical to that described in normal rats: vimentin was confined to smooth muscle cells and glomerular compartment (mensangial cells and podocytes), and α-SMA to arterial smooth muscle cells [23]. The percentage of positive area for vimentin was increased in IRI when compared to Control and VDD groups. Moreover, VDD+IRI group presented higher vimentin expression than did the other studied groups (Figure 6). A similar profile was found concerning α-SMA expression, even though without statistical difference between IRI and VDD+IRI groups (Figure 7). In addition, vitamin D deficiency may be involved in this cellular phenotypic alteration, since the animals from VDD+IRI groups presented a significant expression of vimentin and a slight increase of α-SMA expression when compared to IRI group (Figures 6 and 7).

### Vitamin D deficiency, VDR and fibrosis formation

**Figure 8:**
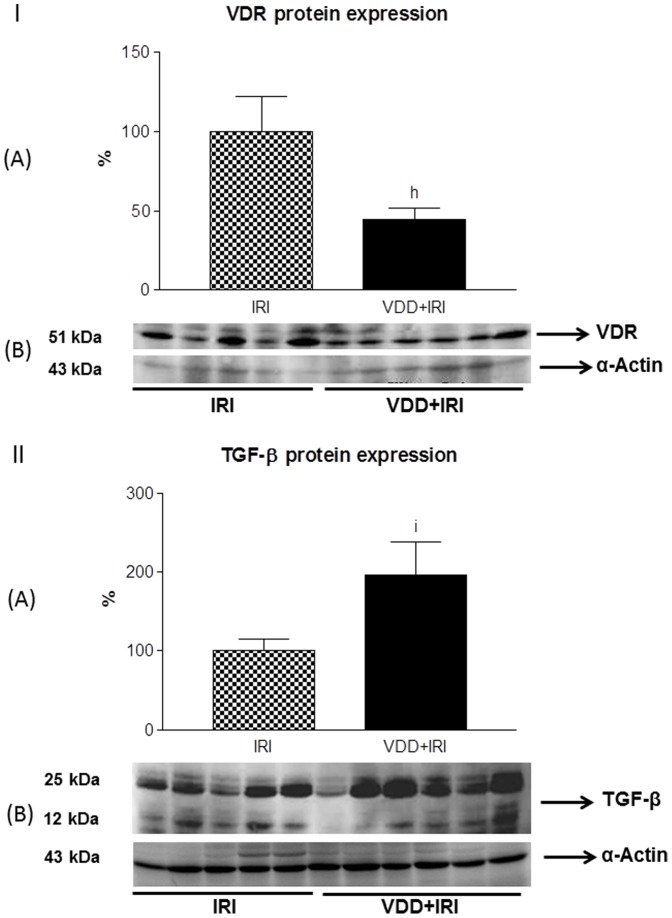
[I] Semiquantitative immunoblotting for VDR in kidney fractions. (A) A densitometric analysis of samples from IRI (n = 5) and VDD+IRI (n = 5) rats is shown. (B) Immunoblots reacted with anti-VDR revealing a 51-kDa band. Values are mean ± SEM. ^h^ p<0.01 vs. IRI. [II] Semiquantitative immunoblotting for TGF-β in kidney fractions. (A) A densitometric analysis of samples from IRI (n = 5) and VDD+IRI (n = 6) rats is shown. (B) Immunoblots reacted with anti-TGF-β revealing a 25 and 12-kDa bands. Values are mean ± SEM. ^i^ p<0.05 vs. IRI.

Our results related to histological and immunohistochemical studies revealed a potential involvement of vitamin D with renal fibrosis. To assess the possible link between vitamin D deficiency and fibrosis formation, we decided to study the role TGF-β expression, the most important pro-fibrotic cytokine, and its relationship with the vitamin D receptor (VDR). For that, we analyzed the renal expression of VDR and TGF-β in both IRI and VDD+IRI groups, since these groups presented expressive percentages of interstitial expansion and fibrosis. We observed a significant decrease in VDR expression (%) in VDD+IRI group (44.3±7.6) when compared to IRI group (100.0±22.0) (Figure 8-I). Concerning TGF-β, we found a significant increased expression (%) of this cytokine in the kidneys of VDD+IRI animals (196.3±41.9) when compared to IRI group (100.1±14.9) (Figure 8-II). So, analyzing the results, we observed that vitamin D deficiency caused a decrease in VDR expression and an increase in TGF-β expression in VDD+IRI group, which had the highest ratio of fibrosis.

## Discussion

In our experimental ischemia/reperfusion injury model, we found renal hypertrophy, increased levels of blood pressure and proteinuria in both ischemic animal groups. Furthermore, we observed enlargement of the interstitial area, including increased infiltration of ED1 positive cells and presence of fibrosis, and phenotypic modification of renal tubular cells. Vitamin D deficiency contributed to the elevation of plasma PTH and decrease of plasma FGF-23 levels as well as for important chronic tubulointerstitial changes. In addition, we found increased expression of cytokine TGF-β1 and decreased expression of VDR receptor and Klotho protein in vitamin D-deficient animals submitted to ischemia/reperfusion injury.

Our results clearly show that animals fed the vitamin D-free diet presented undetectable levels of 25(OH)D. The plasma level of 25(OH)D reflects vitamin D intake from foods and supplements, as well as cutaneous synthesis [24]. In addition to calcium and phosphorus, another important compound related to vitamin D synthesis is PTH level. Our results showed high levels of PTH in VDD, mainly in VDD+IRI group. These alterations were expected since the lack of vitamin D reduces intestinal calcium absorption, leading to a lower level of calcium and higher production of PTH by the parathyroid gland. PTH, in turn, acts on bone tissue in order to attenuate the decrease in serum calcium and the increase in phosphorus excretion [25].

We also investigated the role of vitamin D on blood pressure control. In our study, ischemic and vitamin D deficient rats showed higher levels of blood pressure. This alteration was accompanied by an increased mRNA expression of some RAS compounds, including renin, angiotensinogen and ACE. Furthermore, we found increased levels of plasma aldosterone in VDD, IRI and VDD+IRI groups. So, our data reinforce an important role of vitamin D in blood pressure control. In fact, strong evidences from studies conducted in humans and animals show that vitamin D can be related to a decrease in the renin-angiotensin activity [26], [27]. Also, it has been demonstrated that vitamin D deficiency can led to an upregulation of the RAS, changes in the endothelium, and vascular smooth cells as well [27]–[29]. Li *et al* [30] demonstrated that VDR knockout mice showed increased renin expression and hypertension, and these changes were suppressed by an analogue of vitamin D.

Studies have shown that vitamin D deficiency is associated with increased prevalence of proteinuria in adult population, a marker of CKD progression [31], [32]. Our results showed a progressive and significant increase of proteinuria among the studied groups (C, VDD, IRI and VDD+IRI, respectively). In addition, it was noteworthy that vitamin D deficiency enhanced proteinuria in VDD and VDD+IRI. However, the mechanisms by which proteinuria leads to reduced levels of vitamin D in the body or vice versa are not fully understood [32]. It is known that vitamin D levels can trigger proteinuria by direct and indirect factors. Through direct cellular effects, low levels of vitamin D induce loss of podocytes and development of glomerulosclerosis, damaging the integrity of the glomerular filtration membrane [33]. Indirectly, vitamin D suppresses renin transcription contributing to a reduction in proteinuria by hemodynamic effects [34].

As previously described, the conversion of vitamin D into biologically active form is tightly regulated by several factors, including FGF-23 [12]. In addition to promote renal phosphate excretion, FGF-23 suppresses the production of vitamin D by inhibition of 1-α-hydroxylase and stimulation of 24-hydroxylase [8], [14]–[16]. Recent findings have been supporting an increasingly and important role of FGF-23 as the initial event in the development of CKD. The first step for that is featured by increased levels of FGF-23 preceding changes in calcium, phosphorus, PTH, or even calcitriol levels [35], that is, its respective regulatory factors [36]. Curiously, in our study we found decreased levels of FGF-23 in both VDD and VDD+IRI groups. According to Rodriguez-Ortiz et al [37], this decreased FGF-23 levels could act as a compensatory response to prevent further reductions in calcitriol levels, which could exacerbate the hypocalcemia already expected by the evolution of CKD. So, a plausible explanation for our results is that we evaluated FGF-23 levels in a very early development of renal disease, without loss of renal function. Moreover, we must consider the diet used in our study for feeding the VDD and VDD+IRI animals. Besides being totally depleted of vitamin D, the diet also presented low levels of calcium and phosphorus, which may have contributed for the low levels of FGF-23 found in VDD and IRI+VDD groups.

An important partnership between FGF-23 and Klotho has been described [17]. Klotho proteins form binary complexes with FGF receptors, increasing Klotho affinity and selectivity for FGF-23 [17]. During kidney disease progression, there is reduced expression of Klotho [38], a finding also confirmed in our study. We found a significant reduction in Klotho expression in VDD, IRI and VDD+IRI groups when compared to Control group. In addition, our data showed that vitamin D alone reduced Klotho expression in VDD animals, followed by a similar profile of gene expression for α-klotho. It is described that renal ischemia/reperfusion injury is related to Klotho deficiency. Ming-Chang Hu *et al* [39], using a murine model, showed that ischemic AKI was able to induce an acute and transient state of Klotho deficiency with recovery levels after seven days of injury. Most important, epidemiological studies have shown that increased levels of FGF-23, PTH and low levels of 1,25(OH)_2_D_3_ are features that precede hyperphosphatemia during progression to CKD. Moreover, such alterations in FGF-23, PTH and vitamin D levels is usually followed by a progressive decrease in secreted Klotho protein in urine of CKD patients [39].

It is known that the pathological syndrome of CKD frequently does not heal but becomes self-sustaining, stimulating further kidney injury, resulting in progression of CKD [40]. In our study, a very relevant result related to morphological changes was found mainly in the kidney of vitamin D-deficient rats. Although no changes in renal function have been noticed, we observed an enlargement of the tubulointerstitial compartment associated with histological alterations (fibrosis, tubular atrophy and dilatation, and inflammatory cell infiltrates). Further, we analyzed the expression of two fibrous ECM components (fibronectin and type IV collagen) and infiltrating ED1 cells. We observed increased renal expression of both ECM markers and ED1 positive cells in VDD, IRI and VDD+IRI groups. Moreover, vitamin D deficiency enhanced the respective expressions of fibronectin, type IV collagen and ED1 cells.

Several studies have shown that the factors of initial injury to renal cell lead to: (a) vascular damage, including platelet aggregation and cytokine release; (b) activation of inflammatory responses with recruitment of neutrophils and monocytes, with subsequent pro-inflammatory cytokines releasing; and (c) fibrotic process, including pro-fibrogenic cytokine releasing such as TGF-β and CTGF (connective tissue growth factor) by macrophages and apoptotic parenchymal cells and activation of collagen-producing cells, among others [41]. These factors of initial injury can be initiated by many insults to the kidney, including toxic, ischemic, endocrine, infectious and immunological diseases [42]. In our case, we must consider two main conditions: endocrine and ischemia/reperfusion insult. As a matter of fact, our results showed morphological alterations associated with increased expression of fibrous ECM components and macrophages, including those observed in VDD group even without ischemic kidney injury. Regardless of the initial insult(s), CKD is characterized by stereotyped kidney injury responses seen pathologically as interstitial fibrosis, tubular atrophy, peritubular capillary rarefaction, inflammation and glomerulosclerosis [43]. Moreover, pathologic deposition of fibrillar collagenous matrix, i.e., fibrosis, results when tissues are damaged and normal wound-healing response persist or become dysregulated, usually in response to sustained or repetitive injury. Therefore, our results allow us to infer that our VDD, IRI and VDD+IRI groups were under hemodynamic and hormonal conditions that contributed to the morphological changes found in the renal tissues.

Attempting to establish a link between fibrosis formation and vitamin D deficiency, we evaluated the expressions of TGF-β and VDR in IRI and VDD+IRI groups, both with more prominent interstitial expansion. We observed that vitamin D deficiency caused a decrease in VDR expression and an increase in TGF-β expression in VDD+IRI group, which had the highest ratio of fibrosis. Thus, our data allowed us to infer that adequate levels of vitamin D could help to slow the renal fibrosis formation. In 2006 Tan × et al [44], using the vitamin D analogue paricalcitol in a model of obstructive nephropathy, showed that paricalcitol treatment was able to suppress the expressions of TGF-β and its respective receptor. In addition, paricalcitol treatment restored the expression of VDR receptor, blocked the epithelial-mesenchymal transition (EMT), and inhibited cell apoptosis and proliferation, showing that vitamin D plays a protective role on cellular integrity against cell injury process [44].

The relation of vitamin D to EMT process and our results concerning index of fibrosis in VDD+IRI group, aroused our interest to study whether renal tubule cells were under phenotypic modification. We observed a significant increase of vimentin expression in IRI and VDD+IRI groups and a similar profile of α-SMA expression, although without statistical difference. Based on that, we considered the involvement of vitamin D deficiency in cellular phenotypic alteration, since the animals from VDD+IRI groups presented more prominent expression of both markers. Xiong et al [45], showed that low expression of VDR in CKD could be a potential mechanism linking inflammation to EMT. It is known that pro-fibrotic effect of inflammation depends partly on EMT process. Such effect was possible by sustained stimulation with inflammatory cytokines (TNF-α or IL-1) on epithelial cells. According to the authors, TNF-α suppressed the expression of VDR in various cell types, and sensitized cells to EMT process induced by TGF-β [45]. In our study, we found reduced expression of VDR and increased expression of TGF-β in VDD+IRI group, supporting the idea that vitamin D deficiency is associated with tubulointerstitial damage and interstitial fibrosis. So, a possible mechanism for that would include an association with inflammatory pathways, suggesting a combination between decreased VDR expression with increased TGF-β1 expression in our animals subjected to ischemia/reperfusion injury.

Based on our data, we can conclude that vitamin D deficiency is an aggravating factor for tubulointerstitial damage and formation of interstitial fibrosis after ischemia/reperfusion injury.
